# Exploring the nexus between environmental taxation, air pollution, and health outcomes in emerging and non-emerging EU countries: a space-time perspective

**DOI:** 10.3389/fpubh.2026.1896504

**Published:** 2026-08-11

**Authors:** Adrian Moroșan, Ștefana Belbe, Diana Marieta Mihaiu, Liliana Bunescu, Diana Elena Vasiu, Florina Maria Tăvală

**Affiliations:** 1 Faculty of Economic Sciences, Lucian Blaga University of Sibiu, Sibiu, Romania; 2 Department of Statistics, Forecasts, Mathematics, Faculty of Economics and Business Administration, Babeş-Bolyai University, Cluj-Napoca, Romania; 3 Centre for Interdisciplinary Data Science, Babeş-Bolyai University, Cluj-Napoca, Romania

**Keywords:** air pollution, emerging countries, environmental taxation, European Union, health outcomes

## Abstract

**Introduction:**

The study analyzed the relationship between environmental taxation, air pollution, and health effects in EU member states using an integrated spatial–temporal approach that captures both internal and cross-country effects, with a comparative focus on emerging and non-emerging EU economies.

**Methods:**

To account for fully flexible spatial spillovers, following the recommendation of J. P. Elhorst, we estimate and compare the dynamic spatial panel SLX (Spatially Lagged X), which incorporates the spatial lag in the independent variables, and SDM (Spatial Durbin Model), the model which combines spatial lags of both the dependent and independent variables.

**Results:**

Pollution and resource taxes are the environmental tax instruments with the strongest impact on reducing air pollution. Their effects are observed not only at the national level, but also in neighboring countries, suggesting the presence of significant spatial spillovers. By contrast, energy and transport taxes have limited or no effect on air pollution because transport demand is relatively inelastic and greener alternatives remain limited. The analysis also confirms a persistent relationship between air pollution and adverse health outcomes, positive, statistically significant direct effects were identified for: greenhouse gases, acidifying gases, and ozone precursors on cardiovascular disease deaths; greenhouse gases, acidifying gases, and PM2.5 on respiratory disease deaths. When comparing the direct and indirect spatial effects in the EU emerging economies (Romania, Bulgaria, Hungary, and Poland) with the rest of EU countries, in both the case of taxes effects on pollution as well as pollution effects on health, the differences between the two groups arise from domestic effects rather than cross-border spillovers, which appear comparable in the EU, the only exception being the case of greenhouse gases effects on cardiovascular mortality, where we find evidence of significant differences between the emerging and non-emerging spatial spillovers.

**Discussion:**

The results highlight the importance of developing differentiated policies at the European Union level and support the need for coordination of environmental policies, given the transboundary nature of pollution and the spatial effects across states.

## 1 Introduction

One well-known worldwide issue that has a significant impact on people’s health is climate change. According to the World Meteorological Organization (WHO), 2025 was among the top three hottest years ever recorded, with a temperature that was roughly 1.43 °C higher than the average for the years 1850–1900. The last 11 years, from 2015 to 2025, were the warmest on record. Under these conditions, we can all see that nations all over the world experience more frequent extreme events that have negative impacts on people’s lives and health. These uncontrollable events put pressure on national budgets to repair the damage caused by these meteorological phenomena and to protect the citizens.

The quantities of CO2 in the atmosphere now are higher than they have been in at least 2 million years, while the concentrations of CH4 and N2O are higher than they have been in at least eight hundred thousand years (1). Several hazards, including increased greenhouse effects, ocean acidification, fluctuations in crop yield, food poverty, and air pollution, are linked to high atmospheric CO2 levels. According to WHO data, 99% of people on the planet breathe air that is more polluted and exceeds WHO guidelines, with low- and middle-income nations seeing the largest exposures (2). By reducing the burden of diseases linked to air pollution and helping to mitigate climate change, policies to minimize air pollution offer a win-win solution for both climate and health.

Health is fundamentally dependent on clean air. According to current estimates, the burden of disease from air pollution is comparable to that of other significant global health concerns, including poor diet and tobacco use. It is commonly acknowledged that the greatest environmental risk to human health is air pollution. Every year, exposure to air pollution results in fatalities and reduced years of healthy living. Air pollution was responsible for 232 million healthy years of life lost globally in 2023 and 7.9 million deaths (1 in 8 worldwide deaths). Of these, 0.47 million fatalities were caused by ozone, 2.8 million by household air pollution, and 4.9 million by exposure to ambient PM2.5 (3).

Air pollution increases morbidity and mortality from different diseases. Air pollutants are linked to asthma and other respiratory diseases, heart disease, stroke, and diabetes. People living with these diseases will incur high costs for the healthcare sector (e.g., hospital days, free or subsidized medicines, free medical services). In these circumstances, governments will need to take measures to reduce the economic burden of air pollution-related costs. Most often, these involve reducing air pollution by imposing additional taxes on polluters, namely energy taxes, transport taxes, pollution taxes, and resource taxes. Environmental taxes can have multiple co-benefits beyond climate change (4), including reduced pollution-related disease incidence and lower healthcare costs. Environmental taxes contribute to the state budget in considerable amounts. For example, according to Eurostat, EU member states alone collected 168697.53 million euros from these taxes in 2023. Each country has its own fiscal strategy for environmental taxes, leading to fiscal disparities not only at the global but also at the regional level. This fiscal disparity is also maintained by other disparities observed at the state level, including population exposure to pollution and the degree of economic and social development, and the most exposed to health inequalities seem to be low- and middle-income countries. Health inequalities refer to differences in the care people receive and the opportunities they have to lead healthy lives (life expectancy, the availability of high-quality health services, the costs of health services, etc.). All these health inequalities are numerically proven by statistical reports. The simplest example is the differences in life expectancy worldwide in 2024: although the average is 73.8 years, the highest is 86 years in Monaco and the lowest is 55 years in Nigeria (5). In these conditions, pollution management becomes a priority and a challenge for all countries worldwide, especially for emerging economies that appear vulnerable in responding to threats to human health and the economy (6).

The countries of the world are interconnected in all respects, and environmental interconnections are characterized by spatial dependence and spatial spillovers. Pollution cannot be restricted to the borders of a state; regardless of the type of pollution, its effects will also be felt in the vicinity, and combating it can only be done at the local, national, regional, and global levels. For example, sulfur dioxide and nitrogen oxide emissions released by large German and Dutch industrial centres are carried by air currents, affecting air quality beyond national borders, or coal-fired power plants and mining activities in southern Poland have often generated increased levels of particulate matter (PM2.5) that cross the borders to eastern Germany and the Czech Republic, reducing air quality. However, the most famous example of pollution spillover is the Chornobyl disaster (1986), which, although it occurred in Ukraine, resulted in a radioactive cloud that crossed the borders of numerous European countries, contaminating the soil and vegetation in Sweden, Poland, Germany, and Romania. From the perspective of positive effects, if a state offers tax incentives for the purchase of electric cars, these contribute to reduced traffic on highways connecting neighboring countries and to lower exhaust gas emissions in border areas and along European transit routes.

Although numerous studies examine the impact of environmental taxation on pollution or the impact of pollution on different public health indicators, an integrated analysis is missing. Most studies analyze these relationships separately, as bilateral studies, while few integrate the full transmission mechanism from environmental taxation to health outcomes through pollution reduction, particularly in a multi-country EU context. The present paper aims to contribute to reducing this literature gap by integrating environmental taxation, pollution, and health outcomes into a spatial analysis. The elements of novelty and added value are amplified by the comparative approach of emerging and non-emerging EU countries.

The main objective of the paper is to identify how environmental taxes indirectly improve population health by reducing pollution in EU countries. In practice, we aim to verify whether environmental taxes are delimited from their role as a source of income for the state budget and can instead be used to improve health outcomes. Furthermore, we propose studying these dimensions within a unified special dynamic framework capable of capturing both direct and cross-border spillover effects across EU countries.

Within the European Union, environmental protection is a central priority of public policy. To ensure a healthy life for its citizens, the EU has established a number of key areas of intervention, including measures to reduce pollution. The European Union has committed to improving air quality and reducing emissions of air pollutants through several legislative initiatives (e.g., Directive 2008/50/EC or Directive 2024/2881), creating a common legislative framework for all Member States and clear objectives for reducing pollution. Moreover, at the level of the 27 states, a series of differences are observed regarding the three dimensions: environmental taxes (e.g., environmental tax revenues as % of GDP are higher in Greece, Croatia, Belgium and lower in Ireland, Romania, Czech Republic), pollution indicators (e.g., PM2.5 concentrations above the EU annual limit value were observed in Italy and the eastern and south-eastern countries, while low levels were recorded in the western countries), and health outcomes (e.g., Sweden, Italy and Cyprus record the highest life expectancy at birth, while Bulgaria, Latvia and Romania record the lowest values). All these regional disparities are integrated into our spatial analysis. The inclusion of EU member states in the analyzed sample is justified by their inclusion in the comp and by their neighbors’ primary statistical data from Eurostat, as well as by the fact that a country’s environmental decision affects neighboring countries. The research questions are the following:

*RQ1*: To what extent do environmental taxes contribute to reducing air pollution levels across EU countries, both directly and through spatial spillover effects?

*RQ2*: How do different forms of air pollution affect cardiovascular and respiratory health outcomes in EU countries over time and across space?

*RQ3*: Are emerging EU economies displaying stronger domestic pollution-health effects than non-emerging EU countries?

The research is based on statistical indicators provided by Eurostat for all 27 Member States analyzed and covers the period 2008–2024. Access to standardized environmental, health, and fiscal indicators enables testing dynamic SLX models and identifying differences across countries and regions. Usually, pollution taxes are seen only as economic instruments (public budget-financing instruments) or ecological instruments (pollution-reduction instruments), but their final impact on population health is little analyzed. The direct effect of environmental taxes is the reduction of pollution, but their indirect effect can be quantified in the decrease in cases of people getting sick due to pollution.

The present study makes a consistent contribution to the literature because it aims to demonstrate the existence of direct and spillover effects between environmental taxes and health outcomes in a spatial context (e.g., an environmental tax can reduce the consumption of pollutants, which leads to improved air quality and reduced exposure of citizens to pollutants, which is followed by a reduction in cases of illness and hospitalization, i.e., a reduction in health spending). The importance of this chain lies in its proposing an integrated, rather than fragmented, approach to environmental taxation, pollution indicators, and health outcomes within a specific dynamic framework, with cognitive implications for academia, the political environment, and the public. Furthermore, the use of dynamic SLX models allows the evaluation of both direct and spatial spillover effects of the analyzed indicators in the EU. Another important element of our study is the comparison of emerging and non-emerging EU economies to identify differences in the relationships among taxation, pollution, and health. Finally, it should be remembered that assessing whether environmental taxation produces measurable public health co-benefits can be treated as a component of the climate-health public debate. The current analysis identifies the indirect benefits of fiscal policy, providing practical utility for stakeholders such as public decision-makers (governments and ministries), hospitals, health insurance companies, environmental and health NGOs, the population, and academia.

The remainder of the paper is as follows: Section 2 presents a review of the most relevant studies in the field; the research methodology and data collection are presented in Section 3; the results and their interpretations are detailed in Section 4; the main conclusions are summarized in Section 5.

## 2 Literature review

### 2.1 Theoretical foundations of environmental taxation: environmental externalities and Pigouvian taxation

The theoretical construction of environmental taxation is based on welfare economics, with the central pillar being the correction of market failures generated by externalities, especially those associated with atmospheric emissions. Pigou A. established in 1932 that, when an economic activity produces a negative utility for a third party (known as a negative externality), such as the degradation of air quality through industrial and transport processes, the market price no longer reflects the real social cost but only the private one, so the mechanism of prices objectively set by the market fails (7). Pigouvian taxation intervenes as an indispensable corrective instrument in this paradigm, as it has the role of “internalizing” these external costs by aligning private marginal costs with social ones (8). By applying a direct monetary penalty on the unit of air pollutant (such as fine particles PM2.5, nitrogen oxides NOx or sulfur dioxide SO2), this fiscal mechanism restores Pareto efficiency, which represents that state of resource allocation in which no person can improve his situation without causing harm or loss of utility to at least another person. By taxing the pollutants generated, the economic equilibrium is forced to “migrate” towards a level of production and consumption that is optimal from the perspective of society. The internalization of negative externalities does not represent a simple collection of revenues, but a reconfiguration of the incentives that govern the investment and consumption decisions of economic agents, transforming “uncharged disservices” into direct financial responsibilities (9). Carbon taxes provide an efficient market mechanism for mitigating climate change, offering both environmental and economic benefits (10).

A crucial stage for the development of this conceptual framework was represented by the formulation of the “double dividend hypothesis” according to which pollution taxation policies can simultaneously generate two distinct categories of benefits: a first dividend, the “green” one, refers to the direct improvement of environmental quality as a result of changing the behavior of polluters, who become motivated to reduce emissions in order to avoid the tax burden (11, 12), while the second dividend, the “blue” or economic one, aims at the efficiency gains obtained by using the revenues collected so that various other forms of taxation, sometimes distorted—such as taxes on labor or capital—are reduced. Some studies have qualified this hypothesis by demonstrating that the effectiveness of the second dividend critically depends on the pre-existing fiscal structure and the transparency of the revenue “recycling” mechanism in a national economy, offering the potential to indirectly finance respiratory health infrastructure or subsidize air filtration technologies (13). In this sense, environmental taxation ceases to be perceived as a simple burden, becoming a catalyst for a revenue-neutral fiscal reform, capable of stimulating both employment and ecological sustainability by reducing public spending on respiratory diseases treatment (14, 15).

In contemporary literature, the Pigouvian approach is frequently contrasted with the Coase theorem, which advocates private bargaining and a clear definition of property rights (16). However, in the case of diffuse and transboundary pollutants, such as fine particulate matter (PM 2.5), the high transaction costs of direct bargaining among millions of affected citizens make private solutions practically impossible, requiring state intervention through centralized fiscal measures (17). The justification for this approach is also reinforced by the “polluter pays principle” (PPP), which has become a legal and political foundation in the European Union for charging for atmospheric degradation (18). The efficiency of the tax design, however, depends on rigorous estimates of marginal damages, a task made difficult by scientific uncertainty about the social cost of carbon and the long-term impacts of pollutants on public health (19). Recent research highlights that even “second-best” taxes, which do not perfectly reflect marginal damages due to data limitations, can act as “technological leverage” by stimulating green innovation and accelerating technical change towards clean energy sources, ensuring a sustainable structural transition (20). Studies reveal that carbon taxation is considerably more effective in reducing polluting energy consumption, and carbon taxation policies are a highly robust and successful macroeconomic tool for discouraging fossil fuel use and incentivizing energy efficiency (21).

A study conducted at the level of 38 OECD countries indicated that environmental taxes are an important tool for reducing pollution, but their effect on reducing CO2 emissions is significant only in the long term, the short-term impact not being visible, the results being valid regardless of the level of development or income of the countries analyzed (22). In addition, the effectiveness of environmental taxes is higher at medium and high levels of emissions but decreases as emissions decline. The Environmental Kuznets Curve (EKC) hypothesis has been confirmed for developed and high-income countries but is less applicable to developing and low-income countries. At the same time, the impact of environmental taxes differs across fossil fuels, significantly reducing coal and oil consumption and encouraging natural gas, considered less polluting, leading to the conclusion that taxing CO2 emissions promotes the adoption of less carbon-intensive energy sources.

### 2.2 Environmental taxes and pollution: empirical evidence

There is a vast literature on the relationship between economic growth and health indicators, with most studies confirming a positive link. However, economic growth can negatively affect lifestyles by increasing stress levels, increasing pollution from increased production, and increasing fuel and energy consumption. Economists approach the relationship among environmental degradation, health spending, taxation, and economic growth from different perspectives.

In 2017, Alper analyzed the effects of carbon taxation, economic growth, urbanization, natural gas, and oil consumption across 18 European countries during 1995–2015. The study found that a 1% increase in environmental taxes led to a 0.9% reduction in carbon dioxide emissions. At the same time, a 1% increase in consumption of natural gas and oil products leads to increases in CO2 emissions of 0.1 and 0.7%, respectively, while a 1% increase in urbanization leads to a decrease in CO2 emissions of 0.9% (23). These results demonstrate that environmental taxes can be a useful tool for environmental protection, along with urban growth. This directs population consumption towards natural gas, which is less polluting than oil, and towards modern non-polluting energy sources. The relationship between CO2 emissions, health expenditure, and economic growth was studied by Çobanoğulları in Turkey for the period 1975–2020 using the Autoregressive Distributed Lag (ARDL) model. The study’s conclusions showed that a 1% economic growth generates an increase between 0.553 and 0.297 of CO2 emissions (24). Furthermore, a 1% increase in health expenditure leads to a 0.124% decrease in CO2 emissions over the long term.

Empirical evidence on the effectiveness of environmental taxes is presented in a 2021 study by Morley. The author examined whether environmental taxes affect energy use and pollutant levels. Environmental taxes and pollution are negatively correlated, but there is no correlation between environmental taxes and energy use (25). The econometric analysis used data collected for the period 1995–2006 from EU countries and Norway, using greenhouse gas emissions as an indicator of pollution and energy consumption measured in tons of oil equivalent. Environmental taxes were measured as a percentage of GDP and, respectively, as a percentage of total tax revenues.

In 1995, Grossman and Krueger analyzed the relationship between economic growth, and environmental quality, using panel data from several countries for different types of pollutants (air and water). The authors empirically demonstrate the existence of an “inverted U” relationship (later known as the Environmental Kuznets Curve), meaning that environmental degradation initially increases with economic development in poor countries, but begins to decrease after a certain level of per capita income is reached (26). The paper argues that long-term economic development does not inevitably lead to environmental destruction but can become a path to its improvement. Using an updated, error-corrected database, expanded with ten more years of global air pollution monitoring, in 2002, Harbaugh et al. (27) tested the validity of the inverted U-shape. The results show that this relationship is extremely sensitive to minor changes in the data, the shape of the mathematical functions, or the variables included in the econometric models.

The conventional literature generally suggests that fiscal policy instruments can affect environmental pollution in the long run. But studies show that renewable energy incentives are the most effective policy instrument for mitigating pollution, and that environmental taxes have negative, statistically significant effects on pollution (28). Empirical results confirm that environmental taxes and green finance play a crucial role in improving air quality and reducing the ecological footprint (29). At the EU level, research shows that environmental degradation tends to increase in the early stages of economic development, but subsequently decreases as countries become more prosperous and implement strict green policies (30). The econometric tests applied by the researchers indicate a bidirectional causal relationship between emissions and taxes, confirming that revenues collected from green taxation are a highly effective public instrument for long-term sustainability in Europe. Other studies on the relationship between environmental taxes and CO2 emissions in European Union countries show that environmental taxes must exceed a critical threshold (e.g., 3.02% of cumulative GDP or 2.20% for energy taxes) to be truly effective (31). Once this level of taxation is exceeded, the impact on pollution reduction goes from insignificant to strongly negative, demonstrating that modest taxes do not deter large polluters. European fiscal policies are a basic tool in decoupling economic growth from environmental degradation.

### 2.3 Pollution and health outcomes

There is general acceptance that air pollution negatively affects population health, while CO2 emissions increase the risk of respiratory and cardiovascular diseases, thus increasing healthcare expenditures and mortality rates. Several main research themes dominate the literature, with a notable interdisciplinary approach oriented towards public policy, focusing on the relationships among environmental taxation, air pollution reduction, carbon emissions control, and the transition to sustainable energy. Approaches to public health, mortality, and willingness to pay show the field expanding beyond economics towards the health and behavioral sciences. The relationship between health, pollution, and the economy is dynamic and independent: economic growth can improve health, but it may also intensify pollution.

The study by Zaidi and Saidi (32) examines how economic growth and air quality affect health in Sub-Saharan African countries, using annual data from 1990 to 2015. Their results indicated that health expenditure is positively influenced by economic growth and negatively by CO2 and nitrogen oxides emissions. Dhrifi A. examined how environmental degradation can affect health and the roles of institutions and economic development in mitigating these effects. The study was conducted on a panel of 45 African countries, over the period 1995–2015, using the GMM technique (33). The results indicated a negative relationship between environmental degradation and health, on the one hand, and a positive relationship between the quality of institutions and health, on the other.

The best-known global analysis on the total impact of pollution on mortality is the one coordinated by Landrigan. The study demonstrated that pollution was responsible for approximately 9 million premature deaths in 2015, i.e., 16% of total deaths worldwide, and diseases caused by pollution (heart disease, stroke, lung cancer) disproportionately affect low- and middle-income countries (34). Peters et al. systematically reviewed papers about the relationship between air pollution and later cognitive decline and dementia. The authors focused on 13 papers that reported studies conducted in the USA, Canada, Taiwan, Sweden, and the UK. Although the research methods are different, the conclusion is the same: a greater exposure to PM2.5, NO2/NOx, and CO is associated with increased risk of dementia (35). Other research has shown that daily fluctuations in pollution (temporary increases in pollution from 1 day to the next) are directly linked to peaks in hospital admissions and cardiovascular and respiratory mortality. Peters’s work confirms that there is no completely safe minimum threshold below which particle pollution does not have acute harmful effects (36).

Researchers have estimated that pollution has caused more than 2.7 million lost years of healthy life (DALYs) among people in European nations. Adult ischemic heart disorders had the largest morbidity burden for PM2.5, while diabetes was a significantly linked consequence for NO2 (37). Due to high pollution levels, countries in Eastern and Southeast Europe experience the most severe health effects from air pollution. Research shows that lower-income European nations continue to have much higher death rates from heart attacks and strokes as a result of poor air quality caused by small PM2.5 particles, which are related to home heating and transportation (38). The study, led by Strak as part of the European project ELAPSE, looked at the impact on mortality of long-term exposure to air pollution, using data from six European countries over nearly 20 years. The researchers found that the adverse health effects and mortality risk remain significant even at very low concentrations of pollutants (39). Other research shows that European cities that have invested heavily in green corridors have seen a measurable reduction in cardiovascular mortality among nearby residents (40).

A comparative analysis of the incidence of environmentally induced cardiovascular diseases between Western and Eastern European countries shows that hospital admission rates for acute myocardial infarction on smog days are almost three times higher in Eastern European cities than in Western ones (41). In Western Europe, automobile and industrial emissions are the main problems, while in Eastern Europe, residential burning of coal and wood generates alarming concentrations of pollutants. The socio-economic reality makes infant mortality rates from acute respiratory infections in disadvantaged areas of the East three times higher than the average in Western Europe (42). Significant disparities remain between European countries: Eastern European citizens continue to breathe the most polluted air and bear the highest burden of air pollution-related health impacts, with air pollution mortality rates up to several times higher than in Western European countries (42).

### 2.4 Environmental policy and health: integrated perspectives

The convergence of environmental policy and public health represents a fundamental paradigm shift in contemporary governance, evolving from fragmented sectoral analyses towards a holistic approach, integrated into the conceptual frameworks of “One Health” and “Health in All Policies” (HiAP) (43). This integrated perspective starts from the idea that human health cannot be dissociated from the integrity and stability of the Earth’s biological systems. Research on “planetary boundaries” warns that current human activity has already exceeded the safety margins for several critical areas, generating direct and indirect threats to the longevity of the planet’s population, with polluted air currently recognized as the greatest environmental threat to human life in Europe (44). Climate change directly affects a wide range of human conditions, from cardiovascular and respiratory mortality exacerbated by heat waves to the spread of infectious diseases and malnutrition caused by crop failure. The most vulnerable regions of the world to future climate change, including temperate latitudes, are sub-Saharan Africa, areas affected by El Niño, and large urban agglomerations exposed to the heat island effect (45). The core of this integrated perspective is represented by the “transmission mechanism,” a causal pathway through which fiscal policy interventions implemented “upstream,” such as air pollution taxes. Epidemiological studies have unequivocally demonstrated that fine particles (PM 2.5) are responsible for millions of premature deaths annually, penetrating deep into lung tissue and causing systemic inflammation, lung cancer, and chronic exacerbation of asthma, determinants of clinical outcomes observed “downstream” (34). Studies demonstrated that emissions from residential energy (heating and cooking with solid fuels in countries such as India and China) have the largest global impact on mortality. In highly industrialized regions such as the United States, Europe, and East Asia, emissions from agriculture (fertilizers and livestock) were found to be the most important factor in the formation of toxic fine particles (46). In this context, environmental taxes on fossil fuels, industrial emissions, and polluting transport have ceased to be seen exclusively as simple economic instruments for balancing the budget. They are becoming preventive public health policies, capable of significantly reducing the volume of non-communicable diseases by improving the quality of inhaled air (47). Implementing a rigorous regulatory framework through environmental taxes and green incentives reduces climate change by 0.425% (48). Green incentives have a stronger mitigation impact than taxes.

At the European Union level, this integrated perspective has been institutionalized through the strategic pillars of the “European Green Deal” and the “Zero Pollution” action plan. These far-reaching initiatives consider public health and well-being indicators as key tools for assessing the success of any climate change mitigation policy, aiming to reduce premature deaths caused by polluted air by over 55% by 2030, with a focus on aligning air quality standards with the strict recommendations of the World Health Organization (49). Climate change poses an existential and imminent threat to global human health, affecting the stability of life-support systems through extreme weather events, degraded air quality, and disruptions to food and water security. Global warming acts as a risk multiplier, increasing the transmission of infectious diseases (such as vector-borne ones), exacerbating cardiovascular and respiratory diseases, and causing massive population displacement (50). The annual Lancet Countdown reports have consistently highlighted that the health co-benefits derived from the transition to a carbon-neutral economy, including reduced urban pollution and the promotion of sustainable diets, can offset a considerable share of the costs of investing in green technologies (51).

The presentation of collateral benefits is the fundamental argument used to obtain social consensus and public support for environmental taxation. In the absence of clear explanations to the public about these correlations, environmental taxation could be perceived strictly as a punitive measure (52). Overcoming institutional barriers and harmonizing different time horizons between the ecosystem management and population health sectors are conditions for the successful integration of pollution taxation with public health (53). Most European citizens already perceive respiratory diseases as a direct consequence of polluted air, creating a political window of opportunity for implementing more ambitious green taxes. By using advanced methods to correlate macroeconomic data on taxation with high-resolution geospatial maps, researchers can provide robust empirical evidence to guide priority investments in vulnerable regions. Thus, environmental taxes become an effective tool for protecting public health.

In the econometric literature on spatial spillovers, China is highly represented because it offers large, standardized databases at the provincial or prefectural level. For example, a study by Qin et al. (54) analyzes the spatial and temporal relationships between air pollution (PM2.5, sulfur dioxide, and industrial dust) and public health indicators over more than two decades in China. The authors use spatial econometric models to demonstrate that deterioration of air quality in a given region significantly increases the number of medical visits and diagnoses in neighboring areas. The results highlight that environmental risks do not remain locally isolated but generate a spatial spillover effect across administrative boundaries. Another study on China, by Sun and Wang, examines how pollution affects rural residents’ health-related spending. Air pollution increases regional demand for health services, leading to health and economic disparities between regions (55). At the EU level, research focused on NUTS regions (European statistical classification) or on the cross-border impact of urban ecological policies. Stańczyk analyzed inequalities in public health and life expectancy across regions of the European Union and demonstrated the existence of spatial dependence, i.e., the health status of the population in a NUTS region is positively or negatively shaped by the socio-economic and environmental conditions in surrounding regions (56). According to this analysis, health indicators in the EU tend to converge geographically. A comprehensive study conducted by the European Commission’s Joint Research Center assesses the impact of pollution sources on mortality in 857 European cities. The research divides the impact of pollution into three spatial levels: local (within the city), national, and cross-border. The results of the study demonstrate that a considerable proportion of premature mortality from PM2.5 and NO2 in EU cities is driven by pollutants originating beyond their administrative borders, highlighting the need for integrated public health policies at the European level (57). From the perspective of emerging countries, recent research has revealed significant spatial spillover effects in reducing transboundary pollution, showing that decarbonization efforts in an Eastern European country reduce environmental risks in neighboring states. A recent study examined the relationships among economic growth, energy transformation, and environmental performance across Bulgaria, Croatia, Czechia, Estonia, Hungary, Latvia, Lithuania, Poland, Romania, Slovakia, and Slovenia, covering the period 2010–2023 (58). The researchers conclude that fragmented environmental strategies are ineffective due to geographical interdependence, and recommend coordinating health-environmental policies across the entire region.

## 3 Methodology

We constructed a balanced spatial panel dataset consisting of 27 spatial units representing the EU countries, and 5 and 17 to 18 temporal units representing the temporal window between 2008 and 2023 to 2024, depending on the data availability, considering the environmental taxes, pollution indicators, and health outcomes (see Table 1).

**Table 1:** Summary statistics of all variables and data sources.

Indicator name		Year from	Year to	Min	Max	Mean	Median	SD	Data source
Environmental taxes indicators	Energy Tax per Person (Euro)	2008	2024	93.22	1897.36	547.69	487.66	319.66	Authors’ calculations based on Eurostat database statistics
	Pollution Tax per Person (Euro)	2008	2024	0.00	267.96	32.48	15.35	44.71	
	Transport Tax per Person (Euro)	2008	2024	3.62	780.81	157.13	108.78	157.63	
Air Pollution Index and air pollution indicators	Air Pollution Index	2008	2024	2003807.87	9643152.00	4099804.59	3692025.41	1581363.08	Authors’ calculations based on the methodology presented below.
	Greenhouse Gases (kg/capita)	2008	2024	3888459.22	18595060.00	7958637.73	7168617.09	3063311.95	Eurostat database
	Acidifying Gases (kg/capita)	2008	2023	14702.34	340518.00	45643.34	33510.66	47338.77	
	Ozone precursors (kg/capita)	2008	2023	17714.56	316796.70	44664.09	34593.84	47439.78	
	Particulate Matter (PM2.5 kg/capita)	2008	2023	404.60	14702.71	1873.51	1274.74	2019.94	
Health outcomes indicators	Death Respiratory Diseases (number of deaths per 100,000 inhabitants)	2008	2023	27.72	158.13	77.77	76.20	26.63	Eurostat database
	Death Cardiovascular Diseases (number of deaths per 100,000 inhabitants)	2008	2023	163.26	1210.96	486.88	386.50	250.90	Eurostat database

Source: authors’ elaboration.

To create the Air Pollution Index, we followed the Environmental Pressure Indicators methodology used by the European Commission and Eurostat. The air pollutants indicators we considered are included in three standard EU pressure themes: Climate Change (including Greenhouse gases—GHG), Acidification (including Acidifying Gases), and Air Quality (including Ozone and PM 2.5). To create a single dependent variable, the Pollution Index, we aggregate the air pollutants indicators using Life Cycle Assessment (LCA) weighting factors provided by the EU’s Joint Research Centre (JRC). According to the EU’s Environmental Footprint methodology, the JRC recommends the following weights for calculating an overall environmental score, presented in Table 2 (59). The EU weighting factors were normalized proportionally out of a total of 100%. So, the formula we used for calculating the Air Pollution Index is:

$$\begin{array}{c} \mathrm{Air}\;\text{Pollution Index}=51.36\%\;*\;\text{Greenhouse Gases}\\ +21.85\%\;*\;\mathrm{PM}\;2.5+15.12\%\;*\;\text{Acidifying Gases}\\ +11.65\%\;*\;\text{Ozone Precursors} \end{array}$$

**Table 2:** Air pollution index construction.

Pressure category	Related air pollutants indicators	EU weighting factor (%)	normalized weight
Climate change	Greenhouse gases (GHG), kg/capita	21.06%	51.36%
Particulate matter	PM 2.5, kg/capita	8.96%	21.85%
Acidification	Acidifying gases, kg/capita	6.20%	15.12%
Photochemical ozone	Ozone precursors, kg/capita	4.78%	11.65%

Source: authors’ elaboration.

We use this composite index when estimating the space–time relationship between the breakdown of the pollution-related taxes and the air pollution (See Section 4.1). This allows us to capture overall environmental pressure in a single metric, which is useful because taxes often target general energy or transport behaviors rather than a single gas. In the next phase, however, when assessing the direct and indirect -so-called “spatial spillover”- effects of air pollution on the deaths caused by respiratory and cardiovascular diseases, we perform a pollutant-by-pollutant analysis to capture their individual legacy effects without interference. The reason is bifold. First, each type of pollutant gas affects the cardiovascular and respiratory systems differently, so individual models are necessary to isolate those specific impacts. Second, we chose this methodological approach because of the high interdependence among the pollutants in our study. So, the composite indicator of air pollution is used to measure the overall pressure on the environment, not to measure the link with health outcomes. Therefore, in its construction, we used the weighting scheme from the European Commission’s Joint Research Center’s Environmental Footprint methodology, rather than the authors’ proposed weights, which would be difficult to justify objectively. Although greenhouse gases carry the greatest weight in constructing the composite indicator, the Air Pollution Index, this should not be interpreted as implying dominance of their effects on health. To avoid this interpretation, in the second stage of the research, when we studied the relationship between air pollution and health outcomes, we estimated separate models for each pollutant, reflecting their individual effects independent of the composite air pollution indicator. Moreover, we examine the effects of past values for each pollutant, as the health outcomes considered are not expected to respond only to immediate same-year pollution exposure. For this, we use a 10-year temporal lag in the independent pollution variables, applied only in the pollution–health analysis. This lag was selected to capture long-term cumulative exposure effects, since deaths caused by cardiovascular and respiratory diseases are more plausibly associated with prolonged historical exposure to air pollution than with short-term fluctuations alone. The pollution–health literature documents both short-term and long-term effects of air pollution, but long-term exposure is especially relevant for mortality and chronic cardiovascular and respiratory outcomes (2, 39, 60–65). Medium-term robustness checks were also done using 3-year, and 5-year pollutant lags (see Supplementary Table S2). Given the reduction in the effective time dimension of the panel, the results are interpreted as temporally ordered pollution–health associations rather than as definitive causal estimates.

We seek evidence of direct and, especially, indirect spatial effects (66) between the pollution taxation and air pollution, and between air pollution and respiratory and cardiovascular health outcomes. To attain the specification of fully flexible spatial spillovers, we use Elhorst’s (67) recommendation and estimate and compare the dynamic spatial panel SLX (Spatially Lagged X), which adds the spatial lag in the independent, and SDM (Spatial Durbin Model), the model which combines spatial lags of both the dependent and independent variables. We consider the temporal dependencies within the same unit by introducing the dynamic term, as we expect past levels of pollution to influence present ones, and past levels of health outcomes to be related to present ones. The coefficient of the spatially lagged dependent variable was not statistically significant across the candidate SDM specifications, with *p*-values exceeding the 10% threshold, and likelihood-ratio (LR) tests did not reject the restriction under which the SDM reduces to the SLX. We, therefore, retained the dynamic SLX as the more parsimonious restricted model and preserved the spatial-spillover channel through the spatially lagged explanatory variables. This, in turn means that we avoided the inclusion of an unsupported endogenous spatial lag in the dependent, following the model-selection and parsimony logic in spatial econometrics, according to which more complex spatial specifications should be reduced when additional spatial-dependence terms are not empirically supported (67–70). The SDM restriction checks are reported in Supplementary Table S1.

The equation for the dynamic SLX with cross-sectional and time-specific effects is:

$$Y_{t}=\tau Y_{t-1}+X_{t}\beta +W\;X_{t}\theta +\mu +\alpha_{t}\iota_{N}+\varepsilon_{t}$$

Here, $Y_{t}$ is the N × 1 vector of the dependent variable at time t, $Y_{t-1}$ is its 1 year temporal lag, therefore, τ captures the serial persistence in the dependent, $X_{t}$ is the matrix of covariates with the coefficient vector β, W is the distance based spatial weights matrix and gives the spatial lag of the independent WX, with its associated coefficient θ .

This, in turn, means that spatial vicinity is estimated using this W and defines the spatial neighborhood structure. The baseline specification uses a row-standardized distance-band matrix constructed in GeoDa from country centroid distances. The distance threshold is 1,055 km and corresponds to the minimum connected distance required to ensure that each EU country in the dataset has at least one neighbour, and to ensure spatial continuity. However, since transboundary pollution pathways may also depend on wind patterns and atmospheric circulation, we assess the sensitivity of the results to alternative spatial weights matrices using wider distance-bands of 1,200 km and 1,400 km, as well as k-nearest-neighbour (KNN) matrices with *k* = 3 and *k* = 4 (see Supplementary Table S3 for more details), which provide connectivity-preserving alternatives to the distance-band specification and have been used in related geospatial applications (71).

The spatial panel data structures are modelled using μ for the vector of cross-sectional fixed effects and $\alpha_{t}$ for the time-fixed effects. Finally, $\varepsilon_{t}$ is the idiosyncratic error term.

In the tax–pollution models, $X_{t}$ refers to the contemporaneous tax variables, while in the pollution–health models, $X_{t}$ is replaced by the lagged pollutant exposure $X_{t-k}$ , where *k* is the selected temporal lag, equal to 3, 5 and 10 depending on the specification. The spatially lagged term W $X_{t-k}$ , is computed from the same lagged pollutant exposure variable.

For the exploratory data analysis and spatial weights construction we used GeoDa (72). The analysis was conducted in R using the spml (73) and pml packages (74) to estimate the dynamic spatial panel models. All code, preprocessed dataset and spatial weights matrices are available under a GitHub repository (75).

The classification of EU emerging countries follows the International Monetary Fund (IMF) official classification, which provides an internationally recognized framework. In 2008, at the beginning of our analysis period, the IMF classified several Central and Eastern European member states as emerging and developing economies. However, over the period 2008–2024, many of these countries moved to “advanced economy” status, a process accelerated by their structural convergence that, for most, occurred simultaneously with their integration into the euro area. Specifically, the Czech Republic and Slovakia were reclassified as advanced economies by the IMF in 2009, followed by Estonia in 2011, Latvia in 2014, Lithuania in 2015 and, relatively recently, Croatia in 2023. We included only Romania, Bulgaria, Hungary, and Poland in this category because they were emerging countries throughout the entire period analyzed, 2008–2024. We made this choice to maintain a stable group of countries to ensure data comparability and a consistent benchmark for comparing emerging and non-emerging EU countries. In addition, if we had considered other countries that had the status of emerging countries for some years during the considered period, but have registered in recent years different levels of development compared to the above-mentioned countries that determined the change of their status, we would have created heterogeneity within the same group of emerging countries and would have affected the clarity of the results.

We used Wald tests to compare the spatial direct and indirect effects of the emerging countries versus the other EU countries. Standard errors and confidence intervals for the ratio are obtained using the delta method with cluster-robust covariance estimates. The group-difference *p*-value tests whether the emerging indirect and, respectively, direct effects differ from the rest.

## 4 Results and discussion

### 4.1 Dynamic SLX on the effects of pollution-related taxes on the air pollution index

**Figure 1:**
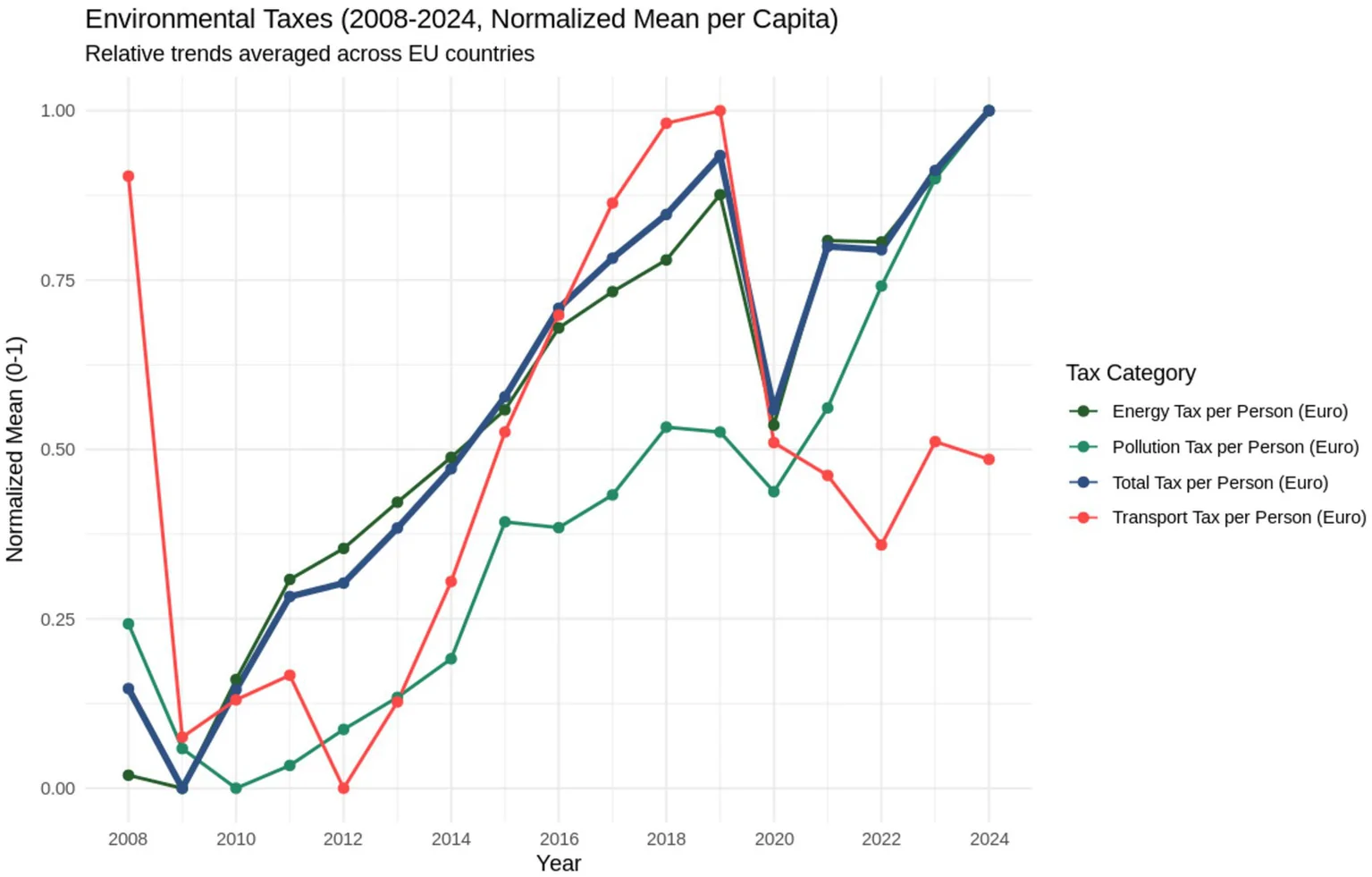
Time series of the environmental taxes. Source: authors’ elaboration.

The objective of the analysis in this section is to determine whether environmental taxes reduce air pollution in EU countries, both directly and through spatial spillover effects, and whether there are significant differences in the results across EU emerging countries compared to the rest of the EU countries. At the EU level, pollution-related taxes are reported under the name of environmental taxes and include energy taxes, transport taxes, and pollution and resources taxes. In Figure 1, the evolution of these taxes during the period 2008–2024 at the level of EU member countries can be observed, with a general trend of growth, though significant inflections in 2020.

**Figure 2:**
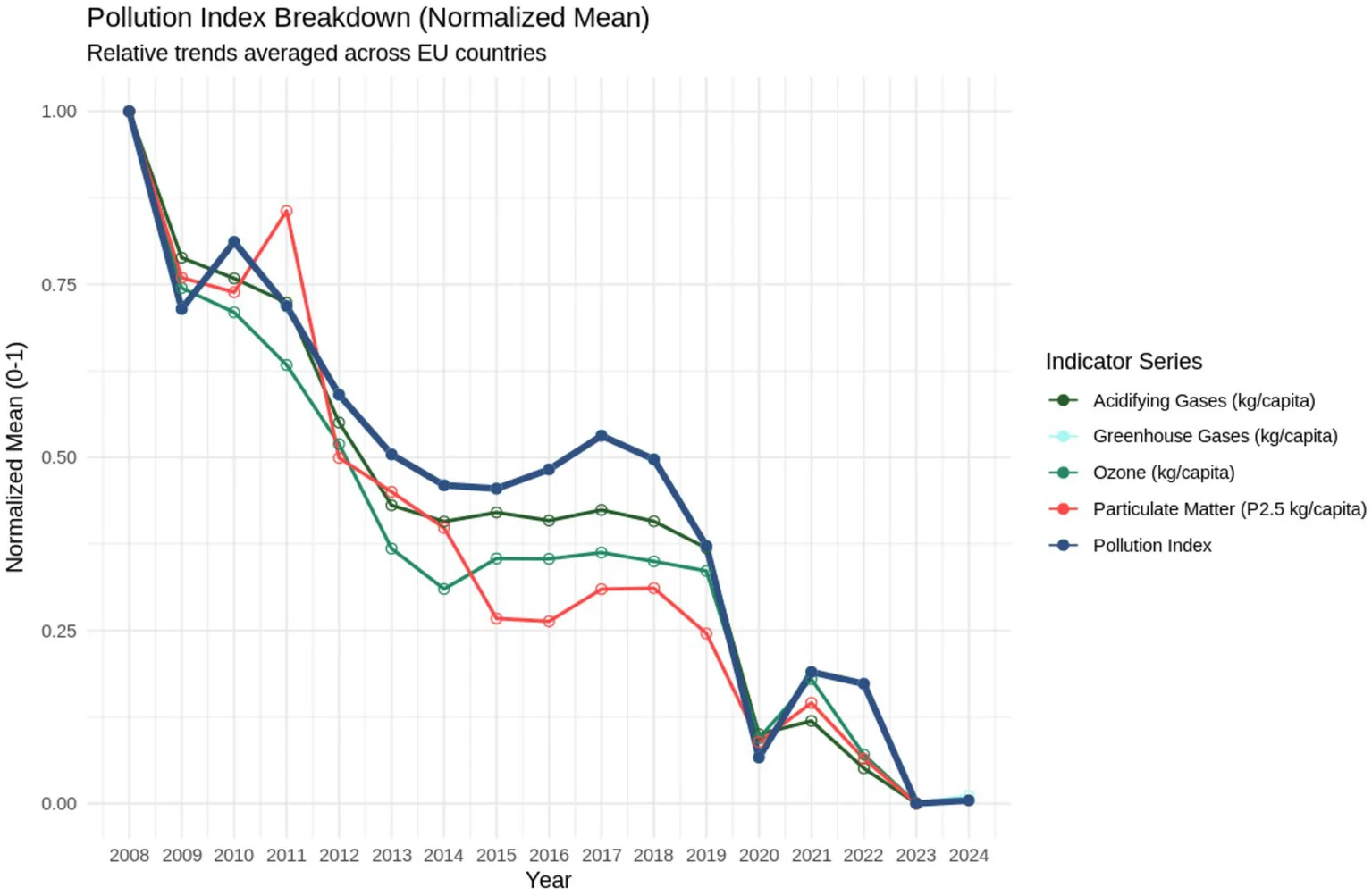
Time series of the Air Pollution Index and its breakdown indicators. Source: authors’ elaboration.

The Air Pollution Index, constructed at the level of EU member states for the period 2008–2024, according to the methodology described above, shows a general downward trend, both overall and in the component indicators, as shown in Figure 2.

**Figure 3:**
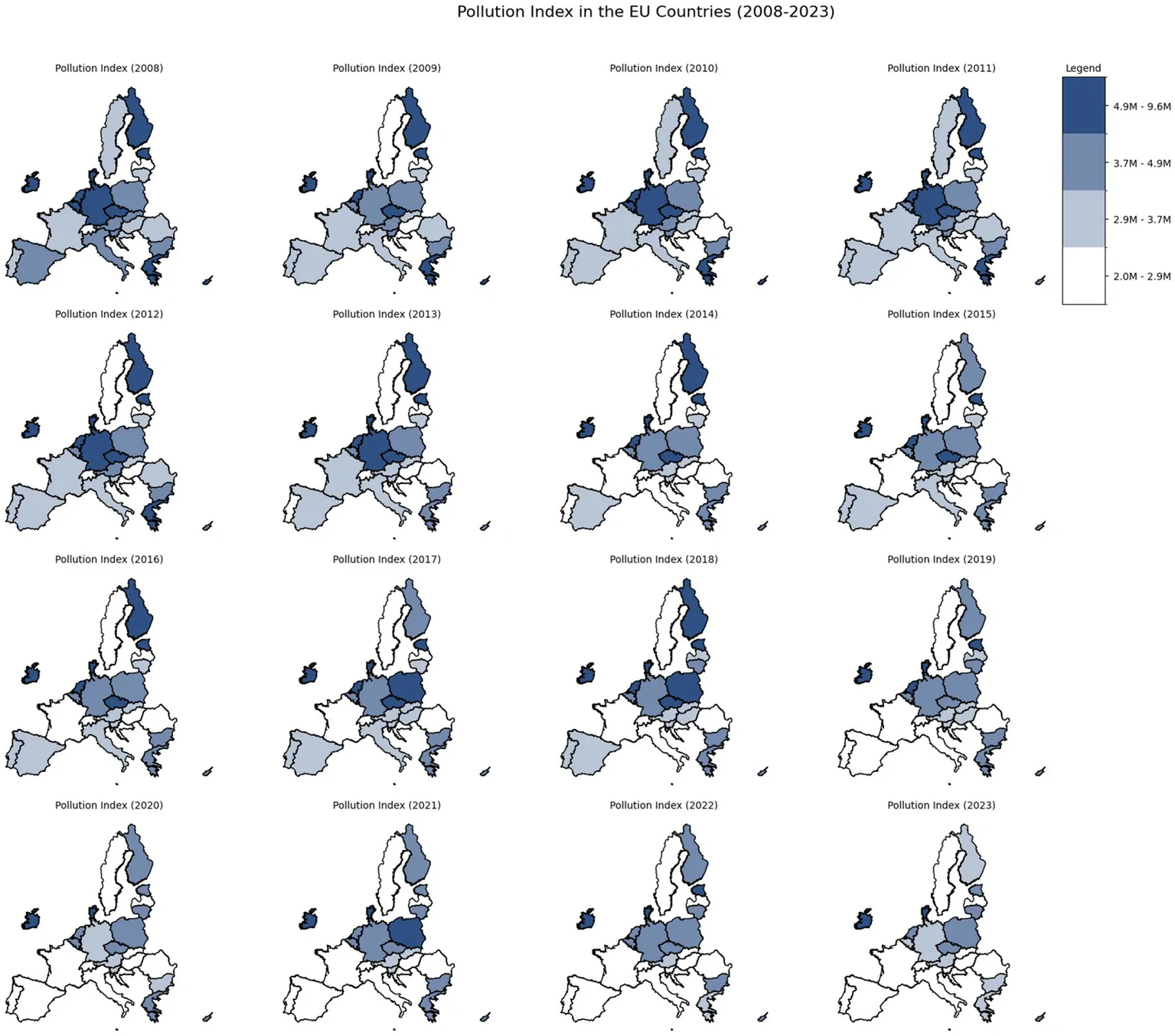
Air pollution index in the EU. Source: authors’ elaboration.

Figure 3 shows the change in the Air Pollution Index for each EU country during the analyzed period. In general, a decrease in pollution intensity is observed across most EU member countries, except for Denmark, Estonia, Ireland, and Luxembourg, which record the highest Air Pollution Index values. These results have different explanations that do not necessarily indicate that they are the most polluting, but are a simultaneous effect of the construction of the aggregate index calculated on per capita indicators, being countries with a small population (see Table 2) and of an intense economic activity and traffic (Luxembourg, Ireland), of a developed industrialized agriculture (Denmark, Ireland), a greenhouse gas emission intensity of electricity generation (Estonia) (76). These results are consistent with studies on the N-shape Environmental Kuznets Curve (EKC), according to which advanced economies may face renewed environmental pressures due to high consumption, industrial and agricultural activities, intensive transport, and energy-intensive digital infrastructures (77, 78).

For modeling the tax effects on pollution, Moran’s I test (79) on the residuals indicates that there is no spatial autocorrelation in the residuals of the SLX model. As per Table 3, pollution is primarily driven by the domestic persistence of the transport and pollution taxes, whilst the energy taxes have no significant direct or indirect effects.

**Table 3:** Dynamic SLX model outputs for the environmental taxes’ effects on the air pollution index.

Energy tax (direct effects)	Transport tax (direct effects)	Pollution tax (direct effects)	W energy tax (indirect effects)	W transport tax (indirect effects)	W pollution tax (Indirect effects)	Lag dependent	Moran I residuals
−0.03 (0.02)	0.10 (0.02)***	−0.01 (0.002)***	0.11 (0.09)	−0.005 (0.07)	−0.01 (0.01)*	0.46 (0.03)***	0.04

Estimate (std. err) ***, **, * significant at 1, 5, 10%.

The spatial-weights robustness checks (Supplementary Table S3) show that the main direct tax–pollution estimates are not driven by the baseline distance-band matrix. The lagged pollution index, the positive direct effect of transport taxes, the negative direct effect of pollution taxes, and the non-significant direct effect of energy taxes remain very similar across alternative spatial weights matrices. The negative indirect effect of pollution taxes is also observed under the alternative distance-band matrices and KNN3, although it becomes weaker under KNN4; therefore, the spatial spillover result is interpreted more cautiously.

At the EU level, environmental taxes consist of:

- Transport taxes, which include taxes on vehicles and transport activity, excluding excise duties on fuel, are generated by vehicle ownership and use. Contrary to conventional expectations, a positive and statistically significant association was found between transport taxes and the air pollution index. This result should be interpreted with caution and does not represent evidence that increasing transport taxes leads to increased pollution. A plausible explanation is reverse causality, meaning that countries with high levels of pollution subsequently adopt higher transport taxes as a public policy measure to discourage the use of polluting vehicles. In these circumstances, the estimated coefficient may reflect both the effect of taxation and the reactive nature of environmental policies. However, behavioral change still responds poorly to higher transport taxes because transport demand is relatively inelastic and green alternatives remain limited, leading to coexistence with increased pollution. This result is also consistent with other recent studies that identified a bidirectional causal relationship between environmental taxes and CO_2_ emission, making reverse causality a plausible explanation for the positive coefficient resulted from our model (30).
- Energy taxes, which include excise duties on fuels, natural gas taxes, coal taxes, electricity taxes, and heating fuel taxes being generated by fuel and energy consumption. The study identified that these taxes in their historical and current form have a weak effect on pollution because their regulatory process is quite outdated at the EU level, since 2003, and were initially designed as taxes to generate revenue for public budgets; they were not designed as taxes with environmental objectives to reduce pollution and decarbonization. This result is also confirmed by the new concern at the EU level, from 2021, to review the EU Energy Taxation Directive (ETD) in accordance with the objectives of the “Fit for 55” package, with the aim that “taxation of motor fuels, heating fuels and electricity in the EU reflects their impact on the environment and on our health” (49). Therefore, this result provides indirect empirical support regarding the need to reform the EU ETD in order to increase its environmental effectiveness.
- Pollution and resources taxes covering emissions to air and water taxes, waste and landfill taxes, and taxes on the extraction of raw materials and use of natural resources. These taxes are the closest to the green tax principle, being directly applied to emissions, they are closer to the Pigouvian principle compared to energy and transport taxes, internalizing externalities. Moreover, there is evidence of significant negative spillover effects of the pollution tax on the pollution index, indicating that the tax reduces pollution in the same country and neighboring countries. Therefore, the negative direct effects suggest that these taxes reduce domestic pollution, and the negative indirect effects suggest that they reduce regional pollution. This result validates the need to coordinate environmental policies in a European spatial context because climate externalities are transnational, the internalization of these externalities produces benefits outside national jurisdiction, and climate policies have regional benefits.

The Lagged Air Pollution Index indicates that past pollution levels are a strong predictor of current pollution levels within the same spatial unit.

Based on the results presented in Table 4, the average spatial spillover tax contribution does not differ significantly between the emerging countries (Romania, Bulgaria, Hungary, and Poland) and the rest of the EU countries. This finding reflects the transboundary nature of air pollution, which does not respect national borders, and therefore supports the need for coordinated European environmental policies, consistent with the results reported by SJ Abbas’ study (80). On the other hand, the average direct, same-country, tax effect on pollution is significantly smaller in the emerging countries. This means that differences between the two groups arise from domestic tax effects rather than cross-border spillovers, which appear comparable in the EU.

**Table 4:** Emerging versus the rest of EU countries dynamic SLX model outputs’ comparison.

Dependent	Emerging	Mean direct effect	Mean indirect effect	Direct effect emerging vs. rest	Direct effect 95% CI	*p*-value direct effect	Indirect effect emerging vs. rest	Indirect effect 95% CI	*p*-value indirect effect
Air pollution index	Yes	0.16	0.61	−0.12	[−0.231, −0.002]	0.05	0.00	[−0.043, 0.043]	1.00
	No	0.28	0.61						

### 4.2 Dynamic SLX on the effects of the air pollution indicators on the health outcomes

The objective of the analysis in this section is to determine whether air pollution worsens health outcomes in EU countries and whether there are significant differences in results between EU emerging countries and the rest of the EU countries. The health outcomes indicators selected for analysis regarding air pollution are cardiovascular disease and respiratory disease death rates. The choice of these 2 dependent variables is sustained by numerous studies demonstrating that air pollution significantly influences respiratory and cardiovascular diseases (60–64).

The sensitivity checks using alternative spatial weights matrices (Supplementary Table S3), indicate that the signs and magnitudes of the coefficients are generally stable across the baseline and wider distance thresholds, and KNN matrices. In a limited number of cases, statistical significance varies around conventional thresholds, but these changes do not alter the substantive interpretation of the results.

For modeling both cardiovascular and respiratory disease deaths, the spatial dependency tests on the residuals for both models are non-significant, suggesting that the spatial autocorrelation has been effectively handled by the SLX model structure. The significant lagged-dependent-variable estimates indicate that past mortality levels are a strong predictor of current mortality within the same spatial unit.

There is a strong temporal persistence given by the 1-year lagged dependent variables across all models (Table 5). The estimate is highly significant, hence, current mortality rates are influenced by the previous year’s health outcomes.

**Table 5:** Dynamic SLX model outputs for the individual past 10 years’ effects of air pollution’s indicators on the health outcomes perceived via the deaths caused by cardiovascular diseases and the deaths caused by respiratory diseases (each row represents a model’s output on a balanced panel: *n* = 27, T = 5, *N* = 135).

Dependent	Independent	Independent lagged (direct effects)	W Independent lagged (indirect effects)	Lag dependent	Moran I residuals
Death cardio-vascular	Greenhouse gases	0.23 (0.21)***	0.57 (0.21)**	0.28 (0.10)***	−0.04
	PM2.5	0.06 (0.04)	0.11 (0.10)	0.39 (0.10)***	−0.05
	Acidifying gases	0.17 (0.04)***	0.15 (0.15)	0.44 (0.09)***	−0.04
	Ozone	0.17 (0.08)**	0.38 (0.30)	0.35 (0.10)***	−0.04
Death respiratory	Greenhouse gases	0.44 (0.21)**	0.1 (0.50)	0.31 (0.08)***	0.03
	PM2.5	0.14 (0.09)*	0.15 (0.21)	0.33 (0.08)***	0.01
	Acidifying gases	0.27 (0.11)**	0.27 (0.38)	0.38 (0.08)***	0.03
	Ozone	0.23 (0.19)	0.18 (0.71)	0.34 (0.08)***	0.05

Estimate (std. err) ***, **, * significant at 1, 5, 10%.

Sensitivity checks using 3-year, and 5-year pollutant lags are reported in Supplementary Table S2.

When modeling the deaths caused by cardiovascular diseases, we identified evidence of positive and statistically significant direct effects in the case of greenhouse gases and acidifying gases. Ozone also shows positive association with deaths caused by these diseases in the baseline specification, although its statistical significance is sensitive to the specification of the structure of the spatial weights matrix. Nonetheless, these results, suggest that historical levels pollution levels contribute to current cardiovascular health issues. These results are supported by previous studies that have documented the impact of air pollution on cardiovascular diseases: the study by Wang et al. (81) on the situation in China reflected that ozone pollution affects the cardiovascular system of people, an aspect confirmed by the World Health Organization (WHO) and by the extensive studies of Gyaase et al. and Tian et al. (82–84); another extensive study conducted by Solomon and Landrigan (85) demonstrated that the increase in greenhouse gases emission causes an increase in the mortality rate from cardiovascular causes, and it was identified that even short-term exposure to sulfur dioxide (SO2), a component of acidifying gases, caused an increase in the number of days of hospitalization due to hypertensive cardiovascular diseases (86).

Interestingly, we also found evidence of spatial spillover effects in the case of greenhouse gases, meaning that past values also influence the cardiovascular health outcomes of the neighboring countries. This result of our analysis, which demonstrates that greenhouse gases from neighboring countries affect cardiovascular mortality in a given country, thus generating cross-country health externalities, is also confirmed by the WHO study (82) which reflects the importance of regional coordination of environmental policies within the EU, as well as by the study conducted by Heo et al. (87) who “*find that air pollution from China significantly increases mortality and morbidity in South Korea (…) and find that a country’s environmental policies could generate substantial hidden benefits for neighboring countries*.”

In the case of the historical effects of the individual pollutant gases upon respiratory deaths, we find evidence of same-country effects for greenhouse gases and acidifying gases, highlighting a long-term legacy on respiratory health. PM2.5 is also positive in the baseline specification, but its statistical significance varies across alternative spatial weights matrices and should therefore be interpreted more cautiously. These results confirm the existence of a cumulative effect of population exposure to air pollution on respiratory diseases, an aspect also confirmed by the specialized literature. In the case of PM2.5, the effects on the respiratory system are among the most documented in the specialized literature, which shows that long-term exposure to PM2.5 causes damage to the respiratory system and significantly increases mortality associated with respiratory diseases (61, 62, 64, 65). Regarding greenhouse gases, they influence both directly the relationship with mortality from respiratory causes (through association with industrial processes and fossil fuel combustion), and indirectly by amplifying ozone and fine particle concentrations in the atmosphere (84). Acidifying gases, especially sulfur dioxide (SO2) and nitrogen oxides (NOx), directly affect the respiratory tract and cause a reduction in lung function (65, 88–90).

Table 6 compares the spatial effects, in the direct and indirect form, of the pollution contributions to the health outcomes between the emerging countries and the remaining EU Member States. Similar to the case of the taxes effects on pollution, here the Wald tests results indicate that differences between the two groups are driven primarily by the direct, same-country, pollution contributions rather than by spatial spillover effects, even if these differences are statistically detectable but numerically low.

**Table 6:** Emerging versus the rest of EU countries dynamic SLX model outputs’ comparison.

Dependent	Independent (10y lag)	Emerging	Mean direct effect	Mean indirect effect	Direct effect emerging vs. rest	Direct effect 95% CI	*p*-value direct effect	Indirect effect emerging vs. rest	Indirect effect 95% CI	p-value indirect effect
Death Cardiovascular	Greenhouse gases	Yes	3.65	9.09	−0.05	[−0.084, −0.008]	0.02	0.05	[0.022, 0.085]	0.00
	Greenhouse gases	No	3.69	9.03						
	PM2.5	Yes	0.42	0.82	−0.01	[−0.033, 0.006]	0.17	0.01	[−0.003, 0.015]	0.23
	PM2.5	No	0.44	0.81						
	Acidifying gases	Yes	1.77	1.63	0.00	[−0.007, −0.002]	0.00	0.01	[−0.004, 0.016]	0.27
	Acidifying gases	No	1.77	1.62						
	Ozone	Yes	1.71	4.09	−0.07	[−0.171, 0.027]	0.15	0.01	[−0.004, 0.024]	0.15
	Ozone	No	1.79	4.07						
Death respiratory	Greenhouse gases	Yes	6.94	1.61	−0.09	[−0.147, −0.029]	0.00	0.01	[−0.035, 0.054]	0.68
	Greenhouse gases	No	7.03	1.60						
	PM2.5	Yes	1.02	1.13	−0.03	[−0.051, −0.016]	0.00	0.01	[−0.01, 0.026]	0.39
	PM2.5	No	1.06	1.12						
	Acidifying gases	Yes	2.85	2.86	−0.01	[−0.012, −0.004]	0.00	0.01	[−0.008, 0.028]	0.27
	Acidifying gases	No	2.85	2.85						
	Ozone	Yes	2.31	1.92	−0.10	[−0.231, 0.035]	0.15	0.00	[−0.016, 0.026]	0.66
	Ozone	No	2.41	1.91						

For cardiovascular mortality, the direct effect is significantly smaller in the emerging group for greenhouse gases and acidifying gases, whereas no significant differences are observed for PM2.5 or ozone. Regarding the indirect effects, only greenhouse gases exhibit a statistically significant difference, while spillover contributions for the remaining pollutants are comparable between the two groups.

For respiratory mortality, significant differences are observed for the direct contributions of greenhouse gases, acidifying gases, and PM2.5, whilst ozone shows no significance in the difference between the two groups. In contrast, none of the indirect contributions differs significantly between the emerging 4 and the remaining EU countries.

Overall, the main differences between the emerging countries and the other EU countries originate primarily from the same-country effects, whereas the estimated effects given by neighboring countries are largely homogeneous across the two groups. Spatial spillovers’ contributions are generally similar, with the exception of greenhouse gases effects on cardiovascular mortality which may reflect differences in emission profiles between emerging and the remaining EU countries. Although cardiovascular mortality remains substantially higher in emerging countries, the estimated direct effects of pollution should not be interpreted as measures of absolute effects upon mortality levels. Rather, they represent the marginal contribution of pollution after accounting for spatial dependence.

**Figure 4:**
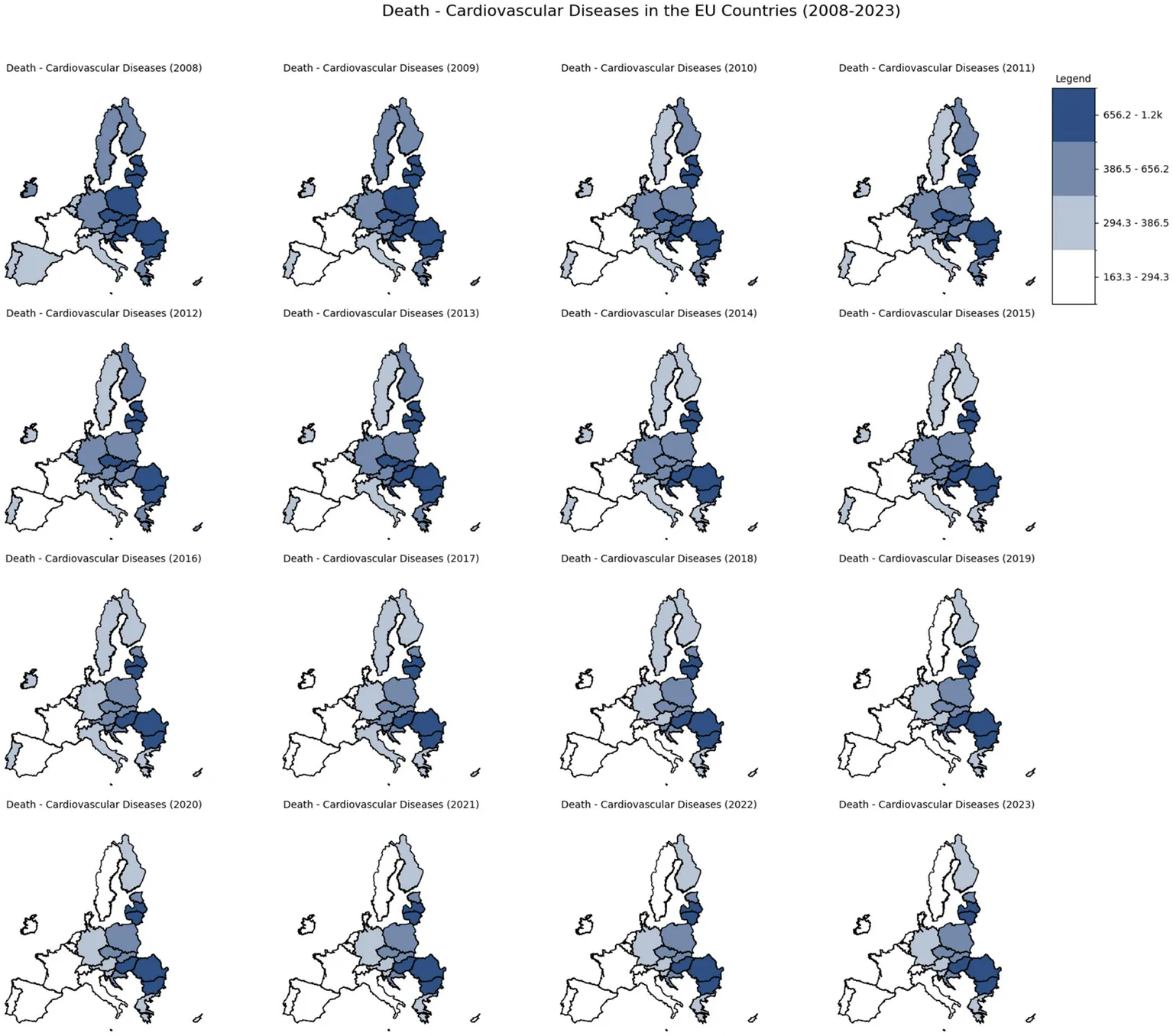
Evolution of deaths caused by cardiovascular diseases in the EU (2008–2023). Source: authors’ elaboration.

Figure 4 above reflects the cardiovascular mortality evolution; emerging countries in Central and Eastern Europe (Romania, Bulgaria, Hungary, and Poland) show higher mortality rates. This result suggests that cardiovascular mortality in these economies is more closely associated with domestic structural factors than by regional spillovers. A possible explanation, although not directly tested by the model, is that these emerging countries often face additional structural vulnerabilities, such as lower quality of health infrastructure, higher socioeconomic inequalities, and lower investment in the prevention and treatment of chronic diseases.

These results highlight the importance of developing differentiated policies at the European Union level. Similar spatial spillover effects support the coordination of European policies to reduce transboundary air pollution. But at the same time, the observed heterogeneity in direct effects indicates that improvements in health outcomes, particularly cardiovascular mortality, in the emerging 4 countries also require domestic structural interventions tailored to each country’s economic and medical realities.

## 5 Conclusion

This study analyzed the relationships among environmental taxation, air pollution, and health effects in EU member states using an integrated spatial–temporal approach that captures both internal and cross-country effects, with a comparative focus on emerging and non-emerging EU economies. The research makes an important contribution to the literature by integrating three dimensions: environmental taxation, pollution, and health into a unified framework, using dynamic spatial panel SLX models that capture both direct and indirect effects, namely cross-border spillover effects. The interest in this topic is justified by the increasingly important role of fiscal instruments in achieving climate and public health objectives at the European level.

Empirical results confirm that environmental taxes have differentiated effects on air pollution. Pollution and resources taxes have proven to be the most effective fiscal instruments that are associated with improvements in air, the most appropriate for the concept of green taxes, both at the national level and in neighboring countries, showing significant spatial effects. Therefore, the negative direct effects suggest that these taxes reduce domestic pollution, and the negative indirect effects suggest that they reduce regional pollution. This result confirms the cross-border nature of environmental problems and highlights the need for closer coordination of fiscal and climate policies at the EU level. In contrast, energy and transport taxes have limited or no effects on air pollution because transport demand is relatively inelastic and green alternatives are still limited; their regulatory process is quite outdated at the EU level, and they were initially designed as taxes to generate public revenues, not as green taxes. Therefore, this result provides indirect empirical support regarding the need to reform the EU ETD in order to increase its environmental effectiveness.

The analysis of the relationship between pollution and health supports the existence of persistent effects on mortality associated with cardiovascular and respiratory diseases. We identified evidence of positive and statistically significant direct effects in the case of greenhouse gases, acidifying gases, and ozone precursors on the deaths caused by cardiovascular diseases. Greenhouse gases generated statistically significant effects, both directly and through spatial propagation mechanisms, indicating that the deterioration of air quality not only affects the population of the state where the emissions are generated but also has consequences for the populations of neighboring regions. In the case of the historical effects of the individual pollutant gases upon respiratory deaths, we find evidence of same-country effects for greenhouse gases, acidifying gases, and PM2.5, highlighting a long-term legacy on respiratory health. In addition, in both situations, the observed effects are manifested in the long term, which confirms the cumulative nature of the risks associated with pollution exposure.

Comparing the direct and indirect effects between the emerging countries (Romania, Bulgaria, Hungary, and Poland) and the remaining EU states shows a pattern: in both modelling phases, environmental taxation—air pollution and air pollution—health outcomes, significant differences between the two groups arise from the same-country effects rather than cross-border, with the exception of greenhouse gases’ effects on cardiovascular mortality where the spatial spillovers are significantly different between the two country groups. Regarding the relationship between environmental taxation and air pollution, no statistically significant differences were found in the spatial spillover effects of environmental taxes, indicating that their cross-border spillover effects on air pollution are similar across the European Union. However, the average direct, same-country, tax effect on pollution is significantly smaller in the emerging countries. This result should be interpreted with caution, given the small size of the group of emerging economies included in the analysis. Although no significant differences were identified in the case of the spatial spillovers, this does not imply that the two groups are structurally similar, since there are significant differences in the direct effects.

A similar pattern emerges in the relationship between air pollution and health outcomes. Wald tests indicate that the differences between the emerging and non-emerging countries are mainly driven by the direct effects of air pollution on the health outcomes within the same countries, while spatial spillover effects are not statistically different. The respiratory and cardiac health of a country is more strongly associated with domestic conditions (such as healthcare system performance, population health status, demographic structure, socioeconomic conditions, and environmental policy implementation) than with differences arising from cross-border spillover effects. Regarding the indirect effects, only greenhouse gases exhibit a statistically significant difference for cardiovascular mortality. These findings suggest that the spatial spillover effects of air pollution are broadly homogeneous across the EU, reflecting the need for coordinated regional policies to combat transboundary pollution. Although cardiovascular mortality remains substantially higher in emerging countries, the estimated direct effects of pollution should not be interpreted as measures of absolute effects upon mortality levels. Rather, they represent the marginal contribution of pollution after accounting for spatial dependence. The higher cardiovascular mortality observed in the emerging economies, together with the relatively similar spillover effects, suggests that in this situation the domestic structural factors may play a more important role than differences in regional spillover effects, highlighting the importance of developing tailored interventions to each country’s economic and medical realities. In addition to regional policies to reduce pollution, domestic measures are also needed to prevent cardiovascular diseases, strengthen the health system and ensure adequate financing, such as: (a) developing national screening and prevention programs for the population in areas with high pollution; (b) investing in medical infrastructure for early diagnosis and treatment of cardiovascular diseases; (c) allocating a portion of environmental taxes to national programs for the prevention and early diagnosis of cardiovascular diseases for people living in areas with high pollution. In this way, environmental taxes could bring benefits both from reducing pollution and improving public health.

The practical implications of the research are relevant to public policymakers, European institutions, ministries of environment and health, tax authorities, and organizations involved in environmental protection and public health. The results support the need for coordination of environmental policies at the European level, given the transboundary nature of pollution and the spatial effects across states. The study also provides empirical arguments for strengthening green fiscal policies within the framework of the objectives of the European Green Deal and of European strategies on pollution reduction and population health protection.

Limitations of the research stem from the fact that: (a) the analysis included only EU member states, not other emerging countries from other regions of the world where climate and health problems may be more pronounced; (b) the relationship between taxation, pollution, and health may also be influenced by other institutional or social factors that may constitute development directions for future research; (c) the existence of the possible endogeneity between transport taxes and air pollution levels. Although the SLX dynamic model includes the lagged dependent variable, which partially reduces the effects of temporal persistence and attenuates some sources of endogeneity, it cannot completely eliminate the possibility of reverse causality; (d) Air Pollution Index is a composite indicator whose construction depends on the adopted framework proposed by the JRC of the European Commission, including the weighting scheme; (e) moreover, the use of a 10-year pollutant lag reduces the effective time dimension of the pollution–health spatial panel. In consequence, these results should be interpreted as temporally ordered associations consistent with cumulative exposure effects rather than as definitive causal estimates.

Future research can: (a) assess the efficiency of public spending on health and the environment, and deepen the analysis to determine whether increases in environmental taxes have contributed to reducing health spending on certain categories of diseases affected by pollution; (b) expand the analysis by including a larger number of emerging economies, including those outside the European Union, to assess the robustness and generalizability of these results.

## Data Availability

Publicly available datasets were analyzed in this study. This data can be found at: https://ec.europa.eu/eurostat/data/database.
