# Supplementary material for: Exploring the nexus between environmental taxation, air pollution, and health outcomes in emerging and non-emerging EU countries: a space-time perspective
Source: Front Public Health. 2026 Aug 11;14:1896504. doi: 10.3389/fpubh.2026.1896504 (PMC13503535; doi:10.3389/fpubh.2026.1896504)
Supplement: Supplementary file 1 [file Data_Sheet_1.PDF]

Supplementary Material

Table S1. Dynamic SDM estimates for the spatially lagged dependent variable

**Analysis block**	**Dependent**	**Independent**	**W Dependent**	**LR between SDM and SLX (1 df)**
Taxes effects on Pollution	Air Pollution Index	Pollution tax	-0.05 (0.08)	0.46
Pollution effects on Health	Cardiovascular deaths	Acidifying gases, 10-year lag	-0.10 (0.52)	0.35
Pollution effects on Health	Cardiovascular deaths	Greenhouse gases, 10-year lag	-0.21 (0.18)	1.46
Pollution effects on Health	Cardiovascular deaths	Ozone, 10-year lag	-0.14 (0.36)	0.67
Pollution effects on Health	Cardiovascular deaths	PM2.5, 10-year lag	-0.13 (0.42)	0.53
Pollution effects on Health	Respiratory deaths	Acidifying gases, 10-year lag	-0.07 (0.64)	0.16
Pollution effects on Health	Respiratory deaths	Greenhouse gases, 10-year lag	-0.04 (0.80)	0.05
Pollution effects on Health	Respiratory deaths	Ozone, 10-year lag	-0.08 (0.60)	0.20
Pollution effects on Health	Respiratory deaths	PM2.5, 10-year lag	-0.06 (0.70)	0.10

Estimate (std. err) ***, **, * significant at 1%, 5%, 10%

The values under Table S1 represent the coefficient attached to the spatially lagged dependent variable in the dynamic SDM alternatives to the SLX and the likelihood-ratio (LR) test values under which the SDM reduces to the SLX. In all cases, the coefficient is statistically insignificant at the 10% level, and the LR test does not reject the SDM-to-SLX restriction. Additional static and dynamic SLX, SDM and SAR checks led to the same conclusion and are available from the authors upon request/reproducible through the accompanying code.

Table S2. Sensitivity checks for the models of the pollution effects over health outcomes using alternative medium-term lags of 3, 5, to the long-term lag of 10 years

Dependent	Independent	Temporal lag	Lag Dependent	Independent lagged (Direct effects)	W Independent lagged (Indirect effects)	AIC	Moran's I residuals
Death Cardiovascular	Acidifying gases	3 years	0.60 (0.05)***	0.01 (0.02)	-0.10 (0.08)	-1806.10	-0.08
Death Cardiovascular	Acidifying gases	5 years	0.60 (0.05)***	0.00 (0.02)	-0.07 (0.09)	-1804.88	-0.07
Death Cardiovascular	Acidifying gases	10 years	0.44 (0.09)***	0.17 (0.04)***	0.15 (0.15)	-897.25	-0.04
Death Cardiovascular	Greenhouse	3 years	0.60 (0.05)***	0.01 (0.04)	-0.03 (0.09)	-1804.32	-0.06
Death Cardiovascular	Greenhouse	5 years	0.59 (0.05)***	0.06 (0.04)	0.07 (0.11)	-1806.10	-0.05
Death Cardiovascular	Greenhouse	10 years	0.28 (0.10)***	0.23 (0.09)**	0.57 (0.21)***	-893.41	-0.04
Death Cardiovascular	Ozone	3 years	0.58 (0.05)***	0.05 (0.03)	-0.04 (0.12)	-1807.57	-0.06
Death Cardiovascular	Ozone	5 years	0.55 (0.05)***	0.10 (0.03)***	-0.08 (0.13)	-1816.97	-0.07
Death Cardiovascular	Ozone	10 years	0.35 (0.10)***	0.17 (0.08)**	0.38 (0.30)	-886.52	-0.04
Death Cardiovascular	P2.5	3 years	0.60 (0.05)***	0.01 (0.02)	-0.01 (0.05)	-1804.22	-0.06
Death Cardiovascular	P2.5	5 years	0.59 (0.05)***	0.04 (0.02)*	0.01 (0.05)	-1808.58	-0.05
Death Cardiovascular	P2.5	10 years	0.39 (0.10)***	0.06 (0.04)	0.11 (0.09)	-884.82	-0.05
Death Respiratory	Acidifying gases	3 years	0.61 (0.05)***	-0.11 (0.06)*	-0.23 (0.21)	-1310.52	-0.06
Death Respiratory	Acidifying gases	5 years	0.60 (0.05)***	-0.10 (0.06)	-0.20 (0.22)	-1309.37	0.01

Death Respiratory	Acidifying gases	10 years	0.38 (0.08)***	0.27 (0.11)**	0.27 (0.38)	-655.53	0.03
Death Respiratory	Greenhouse	3 years	0.63 (0.05)***	-0.04 (0.09)	-0.06 (0.22)	-1306.28	-0.02
Death Respiratory	Greenhouse	5 years	0.62 (0.05)***	0.03 (0.10)	0.24 (0.28)	-1306.93	0.02
Death Respiratory	Greenhouse	10 years	0.31 (0.08)***	0.44 (0.21)**	0.10 (0.50)	-654.08	0.03
Death Respiratory	Ozone	3 years	0.62 (0.05)***	0.00 (0.08)	0.27 (0.32)	-1306.96	0.03
Death Respiratory	Ozone	5 years	0.63 (0.05)***	0.05 (0.08)	0.03 (0.33)	-1306.48	0.00
Death Respiratory	Ozone	10 years	0.34 (0.08)***	0.23 (0.19)	0.18 (0.71)	-649.91	0.05
Death Respiratory	P2.5	3 years	0.62 (0.05)***	-0.06 (0.05)	-0.03 (0.12)	-1308.13	-0.02
Death Respiratory	P2.5	5 years	0.63 (0.05)***	0.01 (0.05)	0.01 (0.14)	-1306.13	0.01
Death Respiratory	P2.5	10 years	0.33 (0.08)***	0.14 (0.09)*	0.15 (0.21)	-651.93	0.01

Estimate (std. err) ***, **, * significant at 1%, 5%, 10%

Table S2 reports sensitivity checks for the models of the pollution effects over health outcomes using alternative medium-term lags of 3, 5, to the long-term lag of 10 years. The lag is applied identically to all pollutants. The spatially lagged independent variable represented by each pollutant separately, is computed using the same lagged exposure measure. Observations with missing values in the dependent variable, lagged dependent variable, lagged independent, or spatially lagged independent are removed before estimation.

Table S3. Sensitivity analysis of the Dynamic SLX estimates to alternative SWM specifications

Dependent	Independent	SWM	Lag Dependent	Independent (Direct effects)	W Independent (Indirect effects)	AIC	Moran's I residuals
Death - respiratory	Acidifying gases	Distance 1,055 km	0.38 (0.08)***	0.27 (0.11)**	0.27 (0.38)	-655.53	0.03
Death - respiratory	Acidifying gases	Distance 1,200 km	0.38 (0.08)***	0.26 (0.11)**	0.18 (0.41)	-655.10	-0.05
Death - respiratory	Acidifying gases	Distance 1,400 km	0.38 (0.08)***	0.28 (0.11)**	0.54 (0.56)	-656.11	-0.03
Death - respiratory	Acidifying gases	KNN 3	0.38 (0.08)***	0.25 (0.11)**	-0.08 (0.25)	-654.98	0.10
Death - respiratory	Acidifying gases	KNN 4	0.38 (0.08)***	0.24 (0.11)**	-0.17 (0.34)	-655.18	-0.01
Death - respiratory	Greenhouse	Distance 1,055 km	0.31 (0.08)***	0.44 (0.21)**	0.10 (0.50)	-654.08	0.03
Death - respiratory	Greenhouse	Distance 1,200 km	0.31 (0.08)***	0.43 (0.21)**	0.01 (0.61)	-654.03	-0.04
Death - respiratory	Greenhouse	Distance 1,400 km	0.31 (0.08)***	0.42 (0.21)**	-0.21 (0.82)	-654.12	-0.02
Death - respiratory	Greenhouse	KNN 3	0.31 (0.08)***	0.36 (0.21)*	-0.44 (0.45)	-655.32	-0.09
Death - respiratory	Greenhouse	KNN 4	0.31 (0.08)***	0.35 (0.21)*	-0.65 (0.54)	-655.93	-0.14
Death - respiratory	Ozone	Distance 1,055 km	0.34 (0.08)***	0.23 (0.19)	0.18 (0.71)	-649.91	0.05
Death - respiratory	Ozone	Distance 1,200 km	0.34 (0.08)***	0.21 (0.19)	-0.03 (0.86)	-649.82	-0.01
Death - respiratory	Ozone	Distance 1,400 km	0.34 (0.08)***	0.21 (0.20)	-0.01 (1.15)	-649.82	-0.01
Death - respiratory	Ozone	KNN 3	0.33 (0.08)***	0.19 (0.19)	-0.25 (0.45)	-650.22	-0.03
Death - respiratory	Ozone	KNN 4	0.34 (0.08)***	0.17 (0.19)	-0.38 (0.51)	-650.56	-0.11
Death - respiratory	P2.5	Distance 1,055 km	0.33 (0.08)***	0.14 (0.09)*	0.15 (0.21)	-651.93	0.01
Death - respiratory	P2.5	Distance 1,200 km	0.33 (0.08)***	0.14 (0.08)	0.09 (0.22)	-651.46	-0.04
Death - respiratory	P2.5	Distance 1,400 km	0.34 (0.08)***	0.14 (0.09)	0.21 (0.31)	-651.83	-0.03
Death - respiratory	P2.5	KNN 3	0.33 (0.08)***	0.13 (0.09)	-0.01 (0.17)	-651.23	-0.05
Death - respiratory	P2.5	KNN 4	0.33 (0.08)***	0.12 (0.09)	-0.06 (0.23)	-651.31	-0.09
Death-cardiovascular diseases	Acidifying gases	Distance 1,055 km	0.44 (0.09)***	0.17 (0.04)***	0.15 (0.15)	-897.25	-0.04
Death-cardiovascular diseases	Acidifying gases	Distance 1,200 km	0.43 (0.10)***	0.15 (0.05)***	-0.01 (0.17)	-895.95	-0.08
Death-cardiovasc	Acidifying gases	Distance 1,400 km	0.43 (0.10)***	0.15 (0.05)***	-0.01 (0.24)	-895.95	-0.09

ular diseases							
Death-cardiovascular diseases	Acidifying gases	KNN 3	0.42 (0.09)***	0.15 (0.04)***	0.12 (0.10)	-897.75	-0.24
Death-cardiovascular diseases	Acidifying gases	KNN 4	0.43 (0.09)***	0.16 (0.04)***	0.19 (0.14)	-898.35	-0.16
Death-cardiovascular diseases	Greenhouse	Distance 1,055 km	0.28 (0.10)***	0.23 (0.09)**	0.57 (0.21)***	-893.41	-0.04
Death-cardiovascular diseases	Greenhouse	Distance 1,200 km	0.32 (0.10)***	0.20 (0.09)**	0.49 (0.26)*	-888.65	-0.09
Death-cardiovascular diseases	Greenhouse	Distance 1,400 km	0.32 (0.10)***	0.19 (0.09)**	0.59 (0.35)*	-887.69	-0.10
Death-cardiovascular diseases	Greenhouse	KNN 3	0.35 (0.10)***	0.21 (0.09)**	0.41 (0.19)**	-890.30	-0.18
Death-cardiovascular diseases	Greenhouse	KNN 4	0.34 (0.10)***	0.19 (0.09)**	0.31 (0.23)	-886.42	-0.11
Death-cardiovascular diseases	Ozone	Distance 1,055 km	0.35 (0.10)***	0.17 (0.08)**	0.38 (0.30)	-886.52	-0.04
Death-cardiovascular diseases	Ozone	Distance 1,200 km	0.36 (0.10)***	0.14 (0.08)*	0.03 (0.36)	-884.28	-0.08
Death-cardiovascular diseases	Ozone	Distance 1,400 km	0.36 (0.10)***	0.14 (0.08)	-0.07 (0.48)	-884.29	-0.09
Death-cardiovascular diseases	Ozone	KNN 3	0.36 (0.10)***	0.17 (0.08)**	0.30 (0.19)	-887.79	-0.18
Death-cardiovascular diseases	Ozone	KNN 4	0.35 (0.10)***	0.17 (0.08)**	0.26 (0.21)	-886.22	-0.11
Death-cardiovascular diseases	P2.5 kg capita	Distance 1,055 km	0.39 (0.10)***	0.06 (0.04)	0.11 (0.09)	-884.82	-0.05
Death-cardiovascular diseases	P2.5 kg capita	Distance 1,200 km	0.40 (0.10)***	0.05 (0.04)	0.05 (0.09)	-883.10	-0.09

Death-cardiovascular diseases	P2.5 kg capita	Distance 1,400 km	0.40 (0.10)***	0.05 (0.04)	0.04 (0.13)	-882.91	-0.10
Death-cardiovascular diseases	P2.5 kg capita	KNN 3	0.41 (0.10)***	0.06 (0.04)	0.10 (0.07)	-885.17	-0.21
Death-cardiovascular diseases	P2.5 kg capita	KNN 4	0.41 (0.10)***	0.06 (0.04)*	0.12 (0.10)	-884.76	-0.12
Pollution Index	Energy tax	Distance 1,055 km	0.46 (0.03)***	-0.03 (0.02)	0.11 (0.09)	-2389.76	0.04
	Pollution tax			-0.01 (0.00)***	-0.01 (0.01)*		
	Transport tax			0.10 (0.02)	0.00 (0.07)		
Pollution Index	Energy tax	Distance 1,200 km	0.46 (0.03)***	-0.03 (0.02)	0.00 (0.10)	-2392.08	-0.01
	Pollution tax			-0.01 (0.00)***	-0.02 (0.01)**		
	Transport tax			0.11 (0.02)	0.13 (0.09)		
Pollution Index	Energy tax	Distance 1,400 km	0.46 (0.03)***	-0.02 (0.02)	-0.07 (0.09)	-2404.30	-0.08
	Pollution tax			-0.01 (0.00)***	-0.04 (0.01)***		
	Transport tax			0.10 (0.02)	0.09 (0.09)		
Pollution Index	Energy tax	KNN 3	0.46 (0.03)***	-0.02 (0.02)	-0.04 (0.05)	-2398.66	-0.14
	Pollution tax			-0.01 (0.00)**	-0.01 (0.00)***		
	Transport tax			0.09 (0.02)	-0.02 (0.04)		
Pollution Index	Transport tax	KNN 4	0.46 (0.03)***	0.10 (0.02)	-0.02 (0.05)	-2391.93	-0.10
	Pollution tax			-0.01 (0.00)**	0.00 (0.01)		
	Energy tax			-0.02 (0.02)	-0.09 (0.06)		

Estimate (std. err) ***, **, * significant at 1%, 5%, 10%

Table S3 reports on the sensitivity analysis of the estimations of the dynamic SLX models using different specifications of the spatial weights matrices (SWM). The preferred distance-band matrix uses the minimum threshold of 1,054.978 km, rounded to approximately 1,055 km, automatically computed by the software used to construct the SWM, GeoDa to avoid isolated countries. Additional matrices use higher distance thresholds of 1,200 km and 1,400 km and k-nearest-neighbour structures (KNN) with k equal to 3 and 4. All matrices are row-standardized. Direct effects refer to the coefficient of the explanatory variable, while indirect effects refer to the coefficient of the spatially lagged explanatory variable. The results show that the signs and magnitudes of the main coefficients' estimates are generally stable across W matrices, with a limited number of coefficients which vary around conventional significance

thresholds. Moreover, as an additional technical check, we compared Euclidean and geographic-distance versions of the baseline distance-band SWM and the neighbour structure and resulting SLX estimates were nearly identical.
